# The Effectiveness of Combined Exercise and Self-Determination Theory Programmes on Chronic Low Back Pain: A Systematic Review and Metanalysis

**DOI:** 10.3390/healthcare12030382

**Published:** 2024-02-01

**Authors:** Alba Navas-Otero, Andrés Calvache-Mateo, Javier Martín-Núñez, Geraldine Valenza-Peña, Sofía Hernández-Hernández, Araceli Ortiz-Rubio, Marie Carmen Valenza

**Affiliations:** Department of Physiotherapy, Faculty of Health Sciences, University of Granada, Av. de la Ilustración 60, 18016 Granada, Spain; albanavas@ugr.es (A.N.-O.); andrescalvache@ugr.es (A.C.-M.); javimn@ugr.es (J.M.-N.); valenzagera@gmail.com (G.V.-P.); sofhdzhdz@gmail.com (S.H.-H.); cvalenza@ugr.es (M.C.V.)

**Keywords:** chronic, disability, exercise, low back pain, quality of life, self-determination theory

## Abstract

Low back pain is a pervasive issue worldwide, having considerable prevalence and a significant impact on disability. As low back pain is a complicated condition with many potential contributors, the use of therapeutic exercise, combined with other techniques such as self-determination theory programmes, has the potential to improve several outcomes. The aim of this systematic review was to explore the effectiveness of combined exercise and self-determination theory programmes on chronic low back pain. This study was designed according to Preferred Reporting Items for Systematic Reviews and Meta-analysis guidelines. A systematic search in three databases (PubMed/MEDLINE, Web of Science, and Scopus) was conducted from September to November 2023. After screening, a total of five random control trials with patients with chronic low back pain were included in this systematic review and meta-analysis. The results showed significant differences in disability (SMD = −0.98; 95% CI = −1.86, −0.09; *p* = 0.03) and in quality of life (SMD = 0.23; 95% CI = 0.02, 0.44; *p* = 0.03) in favour of the intervention group versus the control group.

## 1. Introduction

Back pain is ubiquitous and plagues almost everyone in all countries at some time in their lives (around 20% annually), and, in up to 50% of these, at least once a year [1]. Specifically, lower back pain continues to stand as the primary worldwide contributor to disability, affecting both males and females. It constitutes 7.6% of the total, equivalent to 42.5 million years lived with disability across all age groups [2].

Low back pain has a variable course characterized by often recurrent and transient episodes of pain [3]. Various factors and triggers contribute to instances of back pain, encompassing previous occurrences of back pain, the existence of concurrent chronic conditions, suboptimal mental health, smoking, obesity, and insufficient physical activity levels. These elements are interconnected with overall poorer health, and they are also linked to the onset of episodes of low back pain [4].

How pain is processed, experienced, and understood has a central role in the development and maintenance of disabling pain [5]. The biomedical interpretation of back pain as a medical condition that requires attention and treatment has been shown to increase disability by shifting previous beliefs that back pain is a benign and normal part of everyday life [6]. When considering pain management practices, fear-avoidance theories have become particularly influential in research and in clinical practice. These theories have in common the inclusion of pain education and disability because of an avoidant behavioural style provoked by an excessive fear of pain, movement, and (re)injury. Nevertheless, these theories have been questioned due to observations from clinical practitioners, suggesting the suboptimal identification and assessment of fearful-avoidant patients. Along these lines, it has been recognized that the typical fear-avoidance pattern has its limitations that can be solved with more “personally adapted” strategies.

Along these lines, it is widely accepted that psychosocial prognostic factors should be addressed by clinicians in their assessment and management of patients suffering from low back pain. But, on the other hand, an overview is missing of how these factors are addressed in clinical LBP guidelines.

Health behaviour theories [7] applied in health care have been extensively recommended; specifically, self-determination theory (SDT) and the theory of planned behaviour have become prominent [8]. In particular, these theories have been used to improve our understanding of the response to long-term conditions, help-seeking, decision-making, and intervention uptake [9,10].

SDT posits that motivation for engaging in healthy activities is enhanced when fundamental needs for autonomy, competence, and relatedness are satisfied. Consequently, research in the health domain within the framework of SDT concentrates on how patients perceive practitioners’ support for their autonomy, along with consideration for the other essential psychological needs of competence and relatedness. In the healthcare domain, experimental studies applying SDT typically include healthcare practitioners supporting three basic needs in the patients: autonomy (the feeling of being the originator of one’s behaviours), competence (feeling effective), and relatedness (feeling understood and cared for by others) [11]. Generally, the application of SDT includes a list of SDT-based constructs together with their corresponding definitions, illustrative examples, or questionnaires to assess these constructs.

The focus of SDT is on the quality of generalized motivational orientations that affect behaviour in a specific context [12]. When considering therapies with remarkable acceptance among clinicians, exercise in its different modalities has become very common, due to its moderate effectiveness on pain severity and interference [13]. Furthermore, as low back pain is a complicated condition with many potential contributors, the use of therapeutic exercise combined with other techniques has the potential to result in very different outcomes.

However, to our knowledge, no one study has previously reviewed the characteristics of programmes that combine exercise with the SDT programme’s findings, such as the differences in participants, interventions, comparisons, and results.

With this review, we want to point at the challenges coming with the addition of exercise to self-determination theory programmes in patients with low back pain. Important questions are what, when, and particularly how, studies on patients with low back pain use self-determination theories. In this present paper, we argue that self-determination concepts may help overcome current limitations, providing a short overview of recent theories that have been applied in the context of pain. Finally, we provide a metanalysis of disability and quality of life variables.

## 2. Materials and Methods

### 2.1. Design

A systematic review was conducted according to the Preferred Reporting Items for Systematic Reviews and Meta-analysis (PRISMA) guidelines [14] to explore randomized controlled clinical trials exploring the effectiveness of combined exercise and SDT programmes in people with chronic low back pain. It was previously registered in the PROSPERO (International prospective register of systematic reviews in health and social care), with the ID number CRD42023483640.

### 2.2. Search Strategy

Online research was conducted in the following electronic bibliographic databases: PubMed/MEDLINE, Web of Science (WOS), and Scopus. It was conducted from September to November 2023.

**Figure 1:**
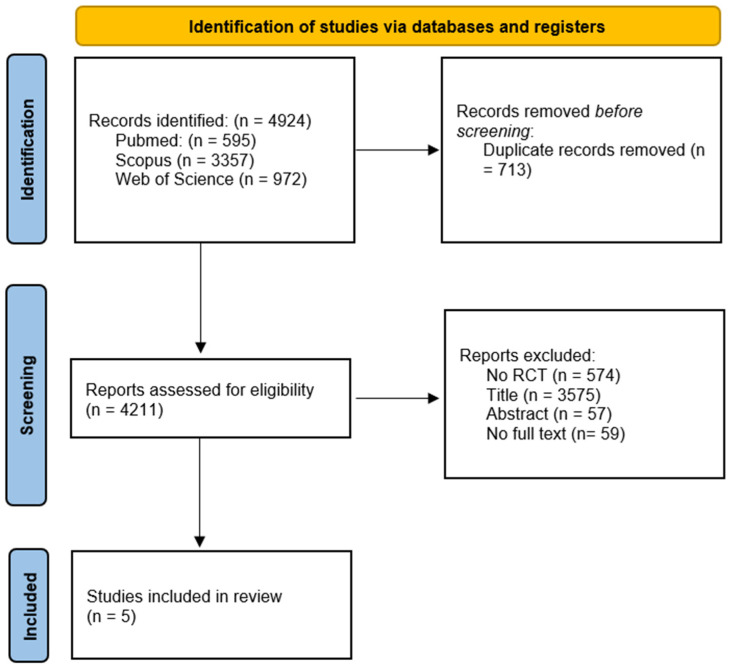
PRISMA flow diagram of the articles through the study selection process.

In order to find relevant studies we used the following search strategy: ((“back pain”) OR (“low back pain”) OR (“lumb* pain) OR (“lumbago”) OR (“backache”)) AND ((“need support”) OR (“autonomy support”) OR (“competence support”) OR (“relatedness support”) OR (“structure”) OR (“involvement”) OR (“motivational climate”) OR (“motivational atmosphere”) OR (“autonomy”) OR (“competence”) OR (“relatedness”) OR (“belonging”) OR (“self-determin*”) OR (“intrinsic motivation”) OR (“intrinsic interest”) OR (“extrinsic motivation”) OR (“autonomous motivation”) OR (“controlled motivation”) OR (“motivation”) OR (“perceived locus of causality”) OR (“Self efficacy”) OR (“self-care”) OR (“self-management”) OR (“patient education”) OR (“sport”) OR (“fitness”) OR (“Exercise therapy”) OR (“Exercise training”) OR (“Exercise program”) OR (“Exercise regime”) OR (“Physical activity”) OR (“Vigorous activity”) OR (“Moderate activity”) OR (“Aerobic exercise”) OR (“Aerobic capacity”) OR (“Aerobic training”) OR (“Resistance training”) OR (“Resistance*program”) OR (“Resistance*regime”) OR (“Resistance*exercise”)). Figure 1 shows the flow details of the studies selected for this review throughout the different phases.

### 2.3. Study Selection

Studies were systematically selected according to our PICOS strategy (participants, interventions, comparisons, outcome, and study design) eligibility criteria: (1) Participants: adults (≥18 years) with chronic low back pain (described as pain lasting more than 3 months); (2) Interventions: combined exercise and self-determination theory programmes; (3) Comparisons: no combined exercise and self-determination theory programmes, no exercise, and usual care or no intervention; (4) Outcomes: pain and disability outcomes; (5) Study design: randomized controlled trials.

To decide the inclusion of studies according to the intervention, we used the definition of self-determination theory provided by Stenberg et al. [15] applied to therapeutic interventions. Additionally, we respected the health-related application of the theory proposed by Ryan et al. [16], which includes the three fundamental needs of autonomy, competence, and relatedness that lead to improved health (e.g., lower pain, anxiety, and higher quality of life), as well as more health-conductive behaviours and improved physical health, referred to as “physical health” hereafter (exercise, ergonomic education, etc.).

To follow those theories, we considered all studies including education and promoting proactivity among the participants, plus exercise.

Records that did not meet the inclusion criteria were removed, as well as duplicates, proceedings, and articles in languages other than Spanish, English or French. Two independent reviewers (ANO and MCV) screened the records retrieved from the search strategy by title and abstracts. After the screening of the title and abstracts, we screened the full texts of the potentially eligible studies. Any discrepancies were resolved through a consensus discussion with a third reviewer not involved in the included studies (AOR). Those who met the inclusion criteria were finally included in the study.

### 2.4. Data Extraction

The following data from the included studies were recorded: author, year of publication, sample size, age (years), gender (percentage of women), disease aetiology, and pain characteristics. Full information is summarized in Table 1. The Downs and Black methodological scale and Cochrane Risk of Bias Tool for randomized trials results are also included. Information about the characteristics of interventions containing experimental group interventions, control group interventions, session duration, frequency, programme duration, outcome instrument, and main results is summarized in Table 2.

When information was lacking or ambiguous, we tried to contact the study’s corresponding author through email. If the data remained unclear or if communication was not possible, we conducted the analysis with the available data. Data extraction was independently conducted by two independent reviewers.

### 2.5. Methodological Quality of the Included Studies

The methodological quality of the included studies was assessed with the Downs and Black quality assessment scale [22]. This scale consists of a 27-item scale with five subscales: reporting (10 items), external validity (3 items), bias (7 items), cofounding (6 items), and power (1 item). The results categorize studies as excellent (26–28), good (20–25), fair (15–19), and poor (≤14) quality. We used the modified version that modified the last item from a 5-point score to a 0- or 1-point score, where a score of 1 is given when a power or sample size calculation was provided and score 0 if there was no information on sample size, power calculation, or clarification regarding the appropriateness of the participant number.

### 2.6. Risk of Bias of the Included Studies

To detect interferences in randomized trials, taking into account participants, comparison groups, and outcomes that can be undetermined by flaws in design, conduct, and analyses, we decided to use a tool for assessing the risk of bias.

The risk of bias was assessed using the Cochrane Risk of Bias Tool for randomized trials (RoB 2) [23]. This tool is structured intro a fixed set of domains of bias, focusing on design, conduct, and reporting. Each domain comprises a set of questions (“signalling questions”) designed to elicit information about trial features pertinent to the risk of bias. An algorithm generates a suggested bias risk judgment for each domain, with possible outcomes categorized as “low”, “high” or “some concerns”.

### 2.7. Statistical Analysis

A meta-analysis was performed using the Review Manager 5 (RevMan 5) software. All the variables included consisted of continuous data. When data were insufficient for meta-analysis purposes, study authors were contacted if it was possible. If contact was not feasible, we used the embedded Review Manager calculator to calculate the missing information when enough data were given (*p*-values or 95% confidence intervals) [24]. To examine statistical heterogeneity, we used the Q statistic and I^2^. We also conducted visual examinations of forest plots to identify outlier studies. The I^2^ delineates the percentage of total variation among studies attributed to heterogeneity rather than chance [25]. An I^2^ over 50–90% was interpreted as an indicator of substantial heterogeneity [26]. The fixed model was used and expressed effects as standardized mean differences (SMD), with confidence intervals when homogeneity was observed.

## 3. Results

### 3.1. Search Selection

Initially, 4924 records were identified from all the databases. Then, 713 duplicated records were removed before screening. After that, 4211 reports were assessed for eligibility. A total of 574 records were excluded, as they did not meet the inclusion criteria specified in our study (no Random Control Trial, no RCT). After screening the titles and abstracts, 3573 records that were clearly unrelated to the theme of this review were also deleted. A total of 59 other records were excluded due to the unavailability of the full text. Finally, 64 records were full-text assessed, with the remaining 5 records being included in the review [17,18,19,20,21]. A PRISMA flow diagram of the articles through the study selection process is shown in Figure 1.

### 3.2. Characteristics of Studies

A total of 5 RCT studies, including 560 participants, were included in this systematic review, from which 421 participants were included in the disability meta-analysis and 347 in the quality-of-life meta-analysis. The majority of the included subjects were women, reaching, in some studies, 71% of the sample. The age range of the participants was between 42 to 71 years old with a diagnosis of chronic low back pain. Of the included studies in this systematic review, 3 had were designed with only two groups (intervention and control), one had 3 groups (2 control groups and one intervention group), and one had 6 groups (3 intervention and 3 control groups).

More information about participant characteristics is shown in Table 2.

All the studies’ interventions were based on a combination of proactive education and exercise, never implemented in isolation. Three of them compared this intervention with usual care, one of them with yoga, and the other two with material-based education. Two of them had a duration of 4 weeks, one a duration of 6 weeks, one a duration of 12 weeks, and the other a duration of 24 weeks. All of them ranged from 4 to 12 sessions with a duration between 30 to 120 min. The instrument used to assess the effect of the intervention in disability were the Roland–Morris Disability Questionnaire and the Oswestry Disability Questionnaire. To assess the quality of life, the studies used the 36-item Short Form Health Survey (SF-36) and EuroQol 5 dimensions (EQ-D5) More information related to the study characteristics, including applied intervention and results, is available in Table 3.

### 3.3. Methodological Quality of the Included Studies

The Downs and Black methodological scale was used to assess the methodological quality of the included studies. The total score of each study is shown in Table 1. Two studies [17,19] obtained a score of 23 points, one [21] obtained a score of 22 points, and the remaining two [18,20] obtained a score of 21 points. This result means that all the studies showed good methodological quality.

### 3.4. Risk of Bias of the Included Studies

**Figure 2:**
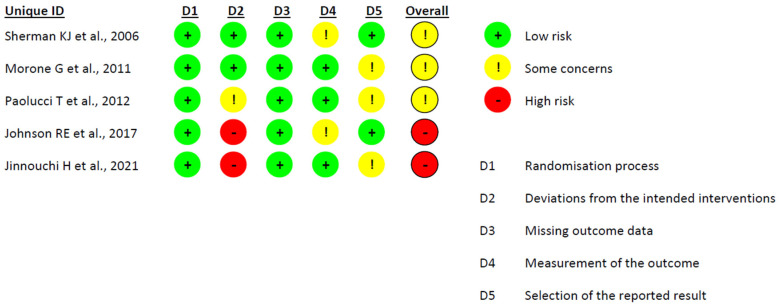
Risk of bias of the included studies [17,18,19,20,21].

Figure 2 shows the risk of bias of each domain and the overall risk of bias evaluated with the Cochrane Risk of Bias Assessment Tool for all the included studies. A high risk of bias was reported by two articles in the second domain [20,21], resulting in an overall high risk of bias. The other three articles showed some concerns in the overall risk of bias. The randomization process domain and missing outcome data domain show low risk of bias in all included studies. The studies of Sherman and Morone et al. [17,18] showed the lowest risk of bias, while the studies of Johnson and Jinnouchi [20,21] had a higher risk of bias.

### 3.5. Meta-Analysis

We considered disability and quality-of-life outcomes for meta-analysis. Four studies were included in the disability meta-analysis, and the other four studies were included in the quality-of-life meta-analysis.

**Figure 3:**
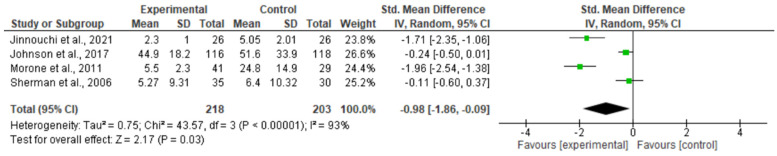
Results of the disability meta-analysis [17,18,20,21].

The disability meta-analysis showed the effectiveness of SDT + exercise interventions in patients with chronic low back pain. Three studies compared the intervention with a material-based intervention, and the other study compared the intervention with usual care. In Figure 3, the results showed significant differences in disability (SMD = −0.98; 95% CI = −1.86, −0.09; *p* = 0.03) in favour of the experimental group. The findings reveal substantial heterogeneity, with an I2 value of 93%.

**Figure 4:**
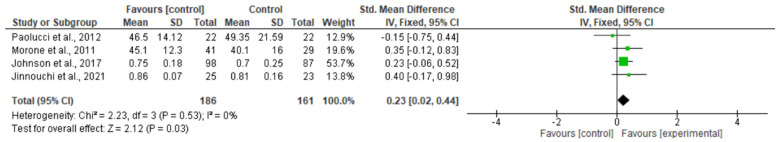
Results of the quality-of-life meta-analysis [18,19,20,21].

In Figure 4, the results show a significant positive effect in the quality-of-life meta-analysis (SMD = 0.23; 95% CI= 0.02, 0.44; *p* = 0.03) in favour of SDT + exercise interventions versus the control group. Two studies compared the intervention with usual care, and the other two compared the intervention with material-based intervention. The findings do not reveal substantial heterogeneity, with an I2 value of 0%.

## 4. Discussion

The objective of this study was to systematically review the effectiveness of combined exercise and self-determination theory programmes in patients with chronic low back pain, and our results provide evidence supporting the effectiveness of those techniques on disability and quality of life. Additionally, these meta-analyses provide support for the use of self-determination-based interventions to achieve immediate benefits for disability and quality of life in patients with chronic low back pain (CLBP).

A total of five studies reporting qualitative data were systematically identified and synthesized into the systematic review. The intervention programmes were wide-ranging and included educational interventions with different supported or non-supported information, combined with different exercise modalities. The studies included were of variable quality and with a high risk of bias. When analysed, the selected papers offered sufficient evidence to support the combination of self-determination theory-based programmes combined with exercise. These results suggest that advocating for patients’ autonomy, now recognized as a crucial healthcare outcome on its own, also contributes to improved mental and physical well-being.

The definition of CLBP determines the pathophysiological processes and behavioural adaptations that coexist during a significant period of time for each individual [27]. Along these lines, the use of compartmental techniques accompanying pharmacological and physical treatments has been extensively proposed and revised in numerous reviews prior [28,29,30]. Our systematic review is the first to consider self-determination theory combined with exercise in the treatment of chronic low back pain, as well as the first to analyse its specific application.

When considering our results related to disability, some behavioural treatments have been proposed as an imperative aspect of chronic low back pain treatment that have to be considered during patient management. This must be due to the restricted functioning of people with disabilities as concerns work, which are also major problems associated with this condition, especially when viewed from the perspective of society. Nevertheless, the large socio-economic burden of LBP associated with disability has become an important treatment goal and outcome measure in research but also an important part of the treatment. Our review combines self-determination theory and exercise; these interventions address psychosocial and motivational factors combined with the training of the body to promote good physical health, which are of importance for analgesic efficacy and reducing disability. Unfortunately, exercise interventions show a large degree of heterogeneity in content and contextual circumstances, and the effectiveness of these interventions can also be dependent on the timing at which the intervention starts. While that aspect still lacks evidence, we found positive results in favour of the combined exercise plus self-determination intervention group when compared to programmes that use only the exercise component [17] or the behavioural component [17,20,21]. These results have not been previously reported in any of the reviews on CLBP patients [31,32,33].

When considering the reported effects of exercise on CLBP patients when combined with SDT-based interventions, a recent systematic review [34] suggests potential underlying mechanisms of action for standard exercise in alleviating disabling chronic low back pain (CLBP) across various domains. These include enhancements in the strength and endurance of back muscles, trunk flexibility, bone strength, blood supply to spinal muscles, joint strength, and intervertebral disc strength, as well as body composition and cardiorespiratory fitness. These improvements are believed to play a role in fostering the healing process within the body’s functions and structures, subsequently resulting in diminished pain and enhanced functionality. In light of the long-term effects of disability, self-determination programmes can target maladaptive thinking and coping strategies to change behaviour and improve mood, being able to provide long-term effects on disability. Unfortunately, our results only provide evidence for short-term effects due to discrepancies between studies as concerns the inclusion of follow-up in their interventions.

On the other hand, our review found significant improvements in quality-of-life outcomes when associated with self-determination and exercise interventions. In addition, those effects are found in studies with short interventions involving exercise coupled with self-determination programmes. Within this type of treatment, exercises used to be associated with pain reduction and quality of life in the short and long term [35,36,37]. Only one of the included studies [19] demonstrated no benefits from self-determination theory combined with exercise on quality of life but found an improvement in disability associated with the same intervention. That study period of treatment was only 4 weeks of back school intervention without supervision of the exercise. A legitimate concern could be that any adverse effects of exercise would not be evident after such a short intervention. One study [21] used a long-term (6 months) intervention of regular tailor-made exercise plus individual consultation, and teaching was associated with a significant improvement in disability and quality of life as compared to those who only received education information through a book.

Furthermore, our review combines behavioural and exercise interventions, which has the strength of having a multidisciplinary profile in which at least one physical dimension (exercise, physical modalities) and one other dimension (psychological or social or occupational) are included in the findings of reported effects on the quality of life.

Strong evidence supports exercise´s beneficial effects on the quality of life and on disability; specifically, Oakman et al. [38], in a recent systematic review, reported that physical conditioning programmes, particularly when combined with cognitive–behavioural interventions, reduced work-related disability when compared with usual care. Contrarily, current evidence indicates that the majority of CLBP patients are used to bed rest and cease many activities because of pain [39,40]. Along these lines, the inclusion of a behavioural component to achieve the resumption of activities while pain persists and to help patients return to more active lifestyles despite persistent LBP seems indispensable. Additionally, the improvement found in disability can be explained by the SDT constructs of perceived autonomy, psychological need satisfaction, and autonomous self-regulation from patients’ perspectives.

The importance of the behavioural component is a determining step for the design and implementation of an intervention plan to legitimise its formulation and ensure that the patient agrees with the formulation. Diverse studies [41,42] have described the priorities and questions that are relevant to patients with back pain; for example, Turner et al. reported that patients with back pain have questions about how to reduce the impact of pain on their lifestyle, while physicians rarely addressed their concerns and were more focused on physical examinations. More recently, a comprehensive article [43] identified several specific recommendations for improving the process to develop the best practices for interview methods, participating in role-playing and receiving feedback to determine the individual needs that be accomplished during treatment.

Behavioural intervention also should be consistent with the patient’s expectations; along these lines, self-determination theories focus on the methods described as a “patient-centred” approach in contrast to a “clinician-centred” approach. Our results are consistent with this debate regarding an expected improvement in CLBP when considering a patient-centred approach on disability and quality of life.

It is essential to recognize certain limitations in this review when interpreting the findings. Firstly, despite employing an extensive range of MeSH terms, including grey literature, and conducting a manual search, there is a possibility that not all relevant studies were identified. Secondly, the diversity of outcome measures is noteworthy, with authors frequently relying on straightforward questions rather than employing validated tools (e.g., patient-reported outcomes) for evaluating these outcomes. Third, the clinical implications for our results can be related to the difficulties in the identification of optimum content and the context of the interventions.

The existing literature shows a clear gap concerning the interplay between the role of self-determination and exercise and their impact on disability and quality of life in individuals with CLBP. This gap is primarily attributable to two key factors. Firstly, the enduring effectiveness and viability of several programmes aimed at managing chronic diseases, such as CLBP, remain unclear. Secondly, the lack of longitudinal studies capable of rectifying the limitations identified in this systematic review further contributes to this gap. Considering these critical aspects in future research is necessary to improve our understanding about self-determination theory plus exercise interventions, as well as their implications for individuals experiencing CLBP over an extended duration.

## 5. Conclusions

Therapeutic programmes combining therapeutic exercise plus self-determination-based theories have a significant effect on disability and quality of life in patients with chronic low back pain. Those programmes show an improvement in those outcomes after 4 to 24 weeks of treatment. On the other hand, this review was not able to compare between modalities, components, and methodologies in the studies included due to the lack of information provided. When considering the clinical applicability of these programmes, the evidence and content have important clinical implications due to the simplicity, multidisciplinarity, and individuality that characterize the intervention proposed. Future studies need to take into account the importance of including long-term follow-up to analyse disability and quality-of-life outcomes.

## Figures and Tables

**Table 1:** Reason for exclusion.

N Reports Excluded	Reason for Exclusion	Explanation
574	No RCT	The type of article was not a randomized controlled trial. They were case studies, non-randomized studies, protocols, etc.
3575	Title	After reading the title, it was verified that it was not related to the topic of the study and/or did not meet any of the PICOS criteria.
57	Abstract	After reviewing the title and being unsure of the possibility of inclusion, the abstract was read. After certifying that it did not meet any of the criteria of our PICO questions, the report was excluded.
59	No full text	For full texts not initially available in the databases consulted, the researchers contacted the authors of the studies to request the full text. When this was not possible and the authors did not respond, the article was excluded.

**Table 2:** Participant characteristics.

Authors, Year	Sample	Sample Age (Years ± SD)	Sex [Women (%)]	Pain Duration	Pain Intensity (VAS/NRS) [Mean (SD)]	Downs and Black	Risk of Bias
Sherman KJ et al., 2006 [17]	101			At least 12 weeks	NR	23	Some concerns
	EG: 35	EG: 42 ± 15	EG: 63%				
	CG1: 36	CG1: 44 ± 12	CG1: 69%				
	CG2: 30	CG2: 45 ± 11	CG2: 67%				
Morone G et al., 2011 [18]	73			At least 3 months		21	Some concerns
	EG: 44	EG: 61.2 (13.3)	EG: 24 (54.5%)		EG: 6.6 ± 2.2		
	CG: 29	CG: 58.6 (12.2)	CG: 21 (72.41%)		CG: 7.1 ± 1.8		
Paolucci T et al., 2012 [19]	100			weeks		23	Some concerns
	EG: 29	EG: NR	EG: 16 (55.1%)		EG: NR		
	EG1:11	EG1: 58.0 ± 13.1	EG1: 5 (45.4%)		EG1: 6 ± 4		
	EG2:18	EG2: 60 ± 15.7	EG2:11 (61.1%)		EG2: 7 ± 2		
			
	CG: 21	CG: NR	CG: 15 (71.4%)		CG: NR		
	CG1(NES): 11	CG1(NES): 56.1 ± 12.9	CG1(NES):7 (63.6%)		CG1:7 ± 2		
	CG2(ES): 10	CG2(ES): 58.4 ± 14.9	CG2(ES):8 (80%)		CG2:8 ± 1		
Johnson RE et al., 2017 [20]	234			100 mm VAS: >20 mm or more RMDQ > 5	EG: 44.9 ± 18.2 CG: 51.6 ± 22.9 VAS	21	High risk
	EG:116	EG: 47.3 ± 10.9	EG:71 (61%)				
	CG:118	CG: 48.5 ± 11.4	IG: 69 (58%)				
Jinnouchi H et al., 2021 [21]	52			At least 3 months	EG: 5.4, 4–7 CG: 5.1, 4–6 NRS	22	High risk
	EG: 26	EG: 65, 62–70	EG: 65.4%				
	IG: 26	CG: 66, 64–71	CG: 61.5%				

Mean ± Standard deviations. Average, Q1–Q3. EG, Experimental Group; CG, Control Group; NRS, numeric reporting scale; VAS, Visual Analogue Scale.

**Table 3:** Study characteristics.

Authors, Year	Experimental Intervention	Control Intervention	Programme Duration	Outcomes	Results	RMDQ Baseline	RMDQ End Treatment
Sherman KJ et al., 2006 [17]	Education (educational talk on proper body mechanics, the benefits of exercise, realistic goal setting, overcoming barriers, and feedback) + exercise (aerobic and strengthening exercises)	CG1: Yoga CG2: Educational book	12 weeks, 12 sessions, 75 min each session	Disability (RMDQ)	Significant intragroup differences; Significant differences compared to CG1 (*p* = 0.034), and no significant differences compared to CG2 (*p* = 0.12)	EG: 9.0 ± 4.1 CG1: 8.1 ± 4.5 CG2: 8.0 ± 4.0	EG: 5.27 ± 9.31 CG1: 3.12 ± 5.6 CG2:6.4 ± 10.32
Morone G et al., 2011 [18]	Back school (theoretical lessons about the anatomical knowledge of the spine and its function and ergonomic positions, pain concepts, psychological aspects, stress management, workplace situations, sport activities, and re-education) + exercise (exercises based on the re-education of breathing, self-stretching trunk muscles, erector spine reinforcement, abdominal reinforcement, and postural exercises)	Usual care (analgesics, myorelaxants, and NSAIDs)	4 weeks, 10 sessions	Disability (ODI), QoL (SF-36)	Significant EG improvements (*p* = 0.018)	EG: 6.6 ± 2.2 CG: 24.8 ± 14.6	EG: 5.5 ± 2.3 CG: 24.8 ± 14.9
Paolucci T et al., 2012 [19]	Back school (education about anatomical information related to the spine, its functioning and ergonomic positions, pain concepts, psychological aspects and stress management, workplace situation, and sport activities) + exercise (exercises based on the re-education of breathing, self-stretching trunk muscles, erector spine reinforcement, abdominal reinforcement, and postural exercises)	Usual care (NSAIDs and myorelaxants)	4 weeks, 10 sessions	Disability (ODI), QoL (SF-36)	Significant EG1 and EG2 improvements (*p* < 0.001)	EG: NR EG1(NES): 24 ± 42 EG2(ES): 28 ± 18 CG: NR CG1(NES): 12 ± 13 CG2(ES): 34 ± 10	EG: NR EG1(NES): 15.64 *p* = 0.001 EG2(ES): 18.28 *p* < 0.001 CG: NR CG1(NES): 2.28 *p* = 0.516 CG2(ES): 3.07 *p* = 0.381
Johnson RE et al., 2017 [20]	Active exercise and education (problem solving, pacing, the regulation of activity, cognitive restoration, feedback, engaging in avoiding certain activities, pacing activities, and hobbies) + educational booklet and audiocassette	Educational booklet and audiocassette	6 weeks, 8 sessions, 120 min each session	Disability (RMDQ), QoL (EQ-D5)	No statistically significant results in reducing disability (−0.6 score; 95% confidence interval, −1.6, 0.4). EG reduced disability by 0.6 points;	EG: 10.6 ± 3.9 CG: 10.9 ± 4.0	EG:44.9 ± 18.2 CG:51.6 ± 22.9
Jinnouchi H et al., 2021 [21]	Brief self-exercise education (100 min consultation, tailor-made self-exercise programme and individualized direct teaching)	Educational book	24 weeks, 4 sessions, 30 min each session	Disability (RMDQ), QoL (EQ-D5)	Improvement on RMDQ −2.3 (−3.3 to 1.3, *p* < 0.001)	EG: 4.7, 1–7 (Average, points) CG: 5.1, 1–9 (Average, points)	EG: 2.3 ± 1 CG: 5.05 ± 2.019

Mean ± Standard deviations. Average, Q1–Q3. CG, Control Group; EG, Experimental Group; EQ-D5, EuroQol 5 dimensions; NSAIDs, nonsteroidal anti-inflammatory drugs; ODI, Oswestry Disability Questionnaire; QoL, Quality of Life; RMDQ, Roland–Morris Disability Questionnaire; SF-36, 36-Item Short Form Health Survey.

## Data Availability

No additional data are available.
